# Mid- to Long-Term Outcomes of Cervical Disc Arthroplasty versus Anterior Cervical Discectomy and Fusion for Treatment of Symptomatic Cervical Disc Disease: A Systematic Review and Meta-Analysis of Eight Prospective Randomized Controlled Trials

**DOI:** 10.1371/journal.pone.0149312

**Published:** 2016-02-12

**Authors:** Yan Hu, Guohua Lv, Siying Ren, Daniel Johansen

**Affiliations:** 1 Department of Spine Surgery, Second Xiangya Hospital of Central South University, Changsha, Hunan, P.R. China; 2 Department of Respiratory Medicine, Second Xiangya Hospital of Central South University, Changsha, Hunan, P.R. China; 3 Orthopaedic Hospital Research Center, Orthopaedic Hospital Department of Orthopaedic Surgery, David Geffen School of Medicine at University of California Los Angeles, Los Angeles, California, United States of America; Universita degli Studi di Palermo, ITALY

## Abstract

**Purpose:**

This study aimed to investigate the mid- to long-term outcomes of cervical disc arthroplasty (CDA) versus anterior cervical discectomy and fusion (ACDF) for the treatment of 1-level or 2-level symptomatic cervical disc disease.

**Methods:**

Medline, Embase, and the Cochrane Central Register of Controlled Trials databases were searched to identify relevant randomized controlled trials that reported mid- to long-term outcomes (at least 48 months) of CDA versus ACDF. All data were analyzed by Review Manager 5.3 software. The relative risk (RR) and 95% confidence intervals (CIs) were calculated for dichotomous variables. The weighted mean difference (WMD) and 95%CIs were calculated for continuous variables. A random effect model was used for heterogeneous data; otherwise, a fixed effect model was used.

**Results:**

Eight prospective randomized controlled trials (RCTs) were retrieved in this meta-analysis, including 1317 and 1051 patients in CDA and ACDF groups, respectively. Patients after an ACDF had a significantly lower rate of follow-up than that after CDA. Pooled analysis showed patients in CDA group achieved significantly higher rates of overall success, Neck Disability Index (NDI) success, neurological success and significantly lower rates of implant/surgery-related serious adverse events and secondary procedure compared with that in ACDF group. The long-term functional outcomes (NDI, Visual Analog Scale (VAS) neck and arm pain scores, the Short Form 36 Health Survey physical component score (SF-36 PCS)), patient satisfaction and recommendation, and the incidence of superior adjacent segment degeneration also favored patients in CDA group with statistical difference. Regarding inferior adjacent segment degeneration, patients in CDA group had a lower rate without statistical significance.

**Conclusions:**

This meta-analysis showed that cervical disc arthroplasty was superior over anterior discectomy and fusion for the treatment of symptomatic cervical disc disease in terms of overall success, NDI success, neurological success, implant/surgery-related serious adverse events, secondary procedure, functional outcomes, patient satisfaction and recommendation, and superior adjacent segment degeneration.

## Introduction

Anterior cervical discectomy and fusion (ACDF) is considered the gold standard for the treatment of radiculopathy and myelopathy due to cervical disc disease [1–4]. Although it generally provides good outcomes [5–7], potential risks include pseudoarthrosis [3,8] and acceleration of adjacent segment degeneration [9–11]. Cervical disc arthroplasty (CDA), as a motion-preserving alternative, was introduced to address these adverse events. The biomechanical advantage of CDA has been demonstrated previously that it can maintain segmental range of motion and cervical kinematics, theoretically reducing or avoiding adjacent segment degeneration [12–16]. However, CDA has its own potential disadvantages, such as higher incidence of heterotopic ossification [17–20] and implant migration or subsidence [21–24]. Many investigators have reported RCTs comparing CDA with ACDF for the treatment of symptomatic cervical disc disease [25–41]. However, the findings of these studies are inconsistent. Some studies reported that compared to ACDF, CDA could provide better neurological outcomes and reduce the rate of adjacent segment degeneration [25–34], whereas other studies reported no difference between the two procedures [35–41]. To clarify these ambiguous findings, we performed a meta-analysis of the current literature to compare mid- to long-term efficacy and safety of CDA with ACDF for the treatment of symptomatic cervical disc disease.

## Materials and Methods

### Search Strategy

The study was conducted following the Preferred Reporting Items for systematic Reviews and Meta-analysis (PRISMA) guidelines [42]. Medline, Embase, the Cochrane Central Register of Controlled Trials databases were searched through January 2016 by using the following key terms: “cervical disc arthroplasty”, “fusion”, “arthrodesis”, and “randomized controlled trial” (S1 Fig). The searches were limited to studies published in English. The reference lists of selected articles and relevant reviews were also reviewed to identify studies not identified in the original search. Two investigators independently reviewed all subjects, abstracts, and the full text of articles that were potentially eligible based on abstract review. Any disagreements between the investigators were discussed and resolved by consensus.

### Eligibility Criteria

We included studies that met the following conditions: (1) prospective randomized controlled trials comparing CDA with ACDF with a minimum 48 months of follow-up; (2) subjects who were older than 18 years of age and had 1-level or 2-level symptomatic cervical disc disease unresponsive to non-operative treatment for at least 6 weeks; (3) at least one desirable outcome should be reported. Articles were excluded if they had any of the following characteristics: (1) non-RCTs, retrospective studies, or case series; (2) follow-up duration was less than 48 months; (3) duplicated publications from the same investigational site.

### Methodological Quality Evaluation

Two reviewers independently performed the quality of the included studies using the 12 criteria recommended by the Cochrane Back Review Group. According to the recommendation by the Cochrane Back Review Group, studies were rated as having “low risks of bias” when at least 6 of the 12 criteria were met without serious flaws. Otherwise, the studies were rated as having “high risk of bias”.

### Data Extraction

Two reviewers independently extracted the following data: study country, publication year, study design, sample size, follow-up duration, patient demographics, prosthesis type, overall success, neurological success, Neck Disability Index (NDI) success, patient satisfaction and recommendation, implant/surgery-related serious adverse events (classified as WHO grade 3 or 4), secondary procedure, NDI, neck and arm pain scores, the Short Form 36 Health Survey physical component score (SF-36 PCS), and adjacent segment degeneration.

### Data Analysis

The analysis was carried out using Reviewer Manager 5.3 software (Cochrane Collaboration, Oxford, UK). For dichotomous variables, the relative risk (RR) and 95% confidence intervals (CIs) were calculated. For continuous variables, the weighted mean difference (WMD) and 95% CIs were calculated. The level of significance was set as P< 0.05. Standard errors, confidence intervals, P values for difference in means, and interquartile ranges were transformed into standard deviation (SD), where necessary, according to the Cochrane Handbook for Systematic Reviews of Interventions. Statistical heterogeneity was evaluated using the chi-square test and Higgin’s I^2^ test. A P value of chi-square test < 0.10 or I^2^ > 50% indicated statistical heterogeneity, prompting a random effects modeling estimate. Otherwise, a fixed effects model was used. Subgroup analysis was performed on patients with only 1-level cervical disc disease.

## Results

### Literature Search

**Fig 1:**
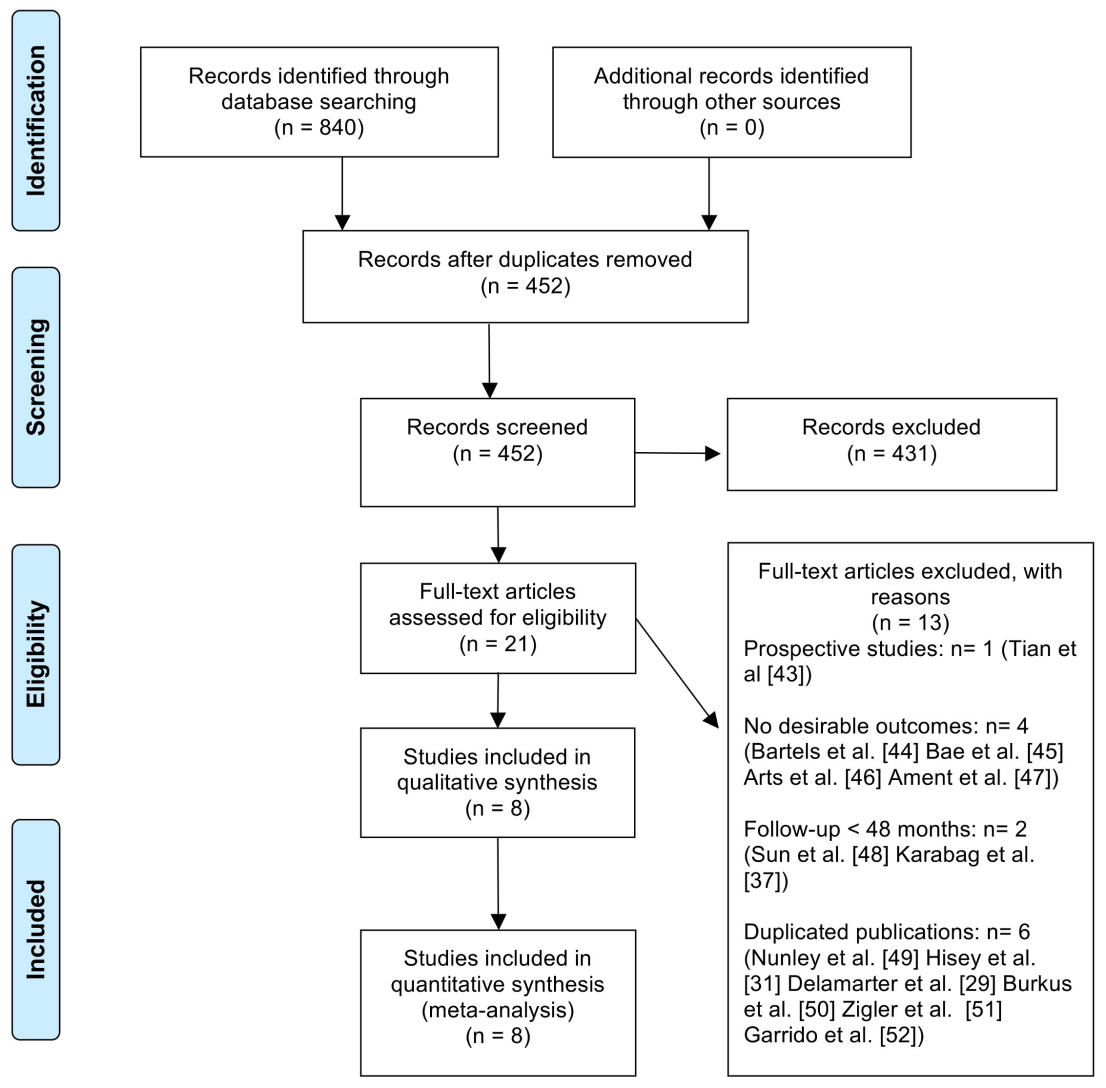
Flow chart showing search strategy.

The details of the literature search and selection are discussed in Fig 1. A total of 840 articles were identified through three electronic database searches. After removal of duplicate and irrelevant articles by title and abstract review, 21 potential articles were retrieved for further full-text evaluation [29, 31, 37, 43–60]. Among them, 13 articles were excluded for not meeting the eligibility criteria [29,31,37,43–52]. Finally, 8 RCTs involving 2368 patients were included in the meta-analysis [53–60]. The basic characteristics of the included studies are shown in Table 1.

**Table 1:** Characteristics of all included studies.

Study	Year	Country	Design	Levels	Enrolled patients		Followed patients		Mean age (years)		Male (%)		Prosthesis	Mean follow-up (months)
					CDA	ACDF	CDA	ACDF	CDA	ACDF	CDA	ACDF		
**Burkus et al**	2014	USA	PRCT, FDA, 31 centers	1	276	265	212	183	43.3	43.9	46.4	46	Prestige	84
**Coric et al**	2013	USA	PRCT, FDA, 1 center	1	41	33	36	27	49.5	49.3	39	43.8	Bryan or Kineflex/C	72
**Davis et al**	2015	USA	PRCT, FDA, 24 centers	2	225	105	202	89	45.3	46.2	50.2	42.9	Mobi-C	48
**Hisey et al**	2015	USA	PRCT, FDA, 23 centers	1	164	81	128	55	43.3	44	47.6	44.4	Mobi-C	48
**Phillips et al**	2015	USA	PRCT, FDA, 24 centers	1	211	184	163	130	45.3	43.7	51.8	51.9	PCM	60
**Sasso et al**	2011	USA	PRCT, FDA, 30 centers	1	242	221	181	138	44.4	44.7	45.5	51.1	Bryan	48
**Zhang et al**	2014	China	PRCT, 11 centers	1	55	56	55	56	44.8	46.7	45.5	46.4	Mobi-C	48
**Janssen et al**	2015	USA	PRCT, FDA, 13 centers	1	103	106	79	73	42.1	43.5	44.7	46.2	ProDisc-C	84

PRCT: prospective randomized controlled trial, FDA: food and drug administration, CDA: cervical disc arthroplasty, ACDF: anterior cervical discectomy and fusion

### Methodological Quality Assessment

The methodological quality of all included studies is presented in Table 2. All eight studies were rated as “low risk of bias” according to the Cochrane Back Review Group criteria. One study [59] failed to clearly report adequate randomization and only one study provided the information of allocation concealment [59]. Blinding of patients, surgeons, and assessors were not considered achieved because of the nature of the studies and evident difference of implant design. Missing information such as the absence of intention-to-treatment analysis and follow-up loss were presented in seven studies [53–58,60].

**Table 2:** Risk of bias assessment of all included studies.

	Burkus et al.	Coric et al.	Davis et al.	Hisey et al.	Phillips et al.	Sasso et al.	Zhang et al.	Janssen et al.
**Adequate randomization**	+	+	+	+	+	+	Unclear	+
**Allocation concealment**	Unclear	Unclear	Unclear	Unclear	Unclear	Unclear	Unclear	+
**Blinding of patients**	-	-	-	-	-	-	-	-
**Blinding of care provider**	-	-	-	-	-	-	-	-
**Blinding of outcome assessor**	-	-	-	-	-	-	-	-
**Acceptable drop-out rate**	Unclear	+	+	-	+	-	+	-
**ITT analysis**	-	-	-	-	-	-	+	-
**Free of selective reporting**	+	+	+	+	+	+	+	+
**Similar baseline**	+	+	+	+	+	+	+	+
**Avoided or similar co-interventions**	+	+	+	+	+	+	+	+
**Acceptable compliance**	+	+	+	+	+	+	+	+
**Similar timing**	+	+	+	+	+	+	+	+
**Total score**	6	7	7	6	7	6	7	7

### Overall Success, NDI Success, and Neurological Success

**Fig 2:**
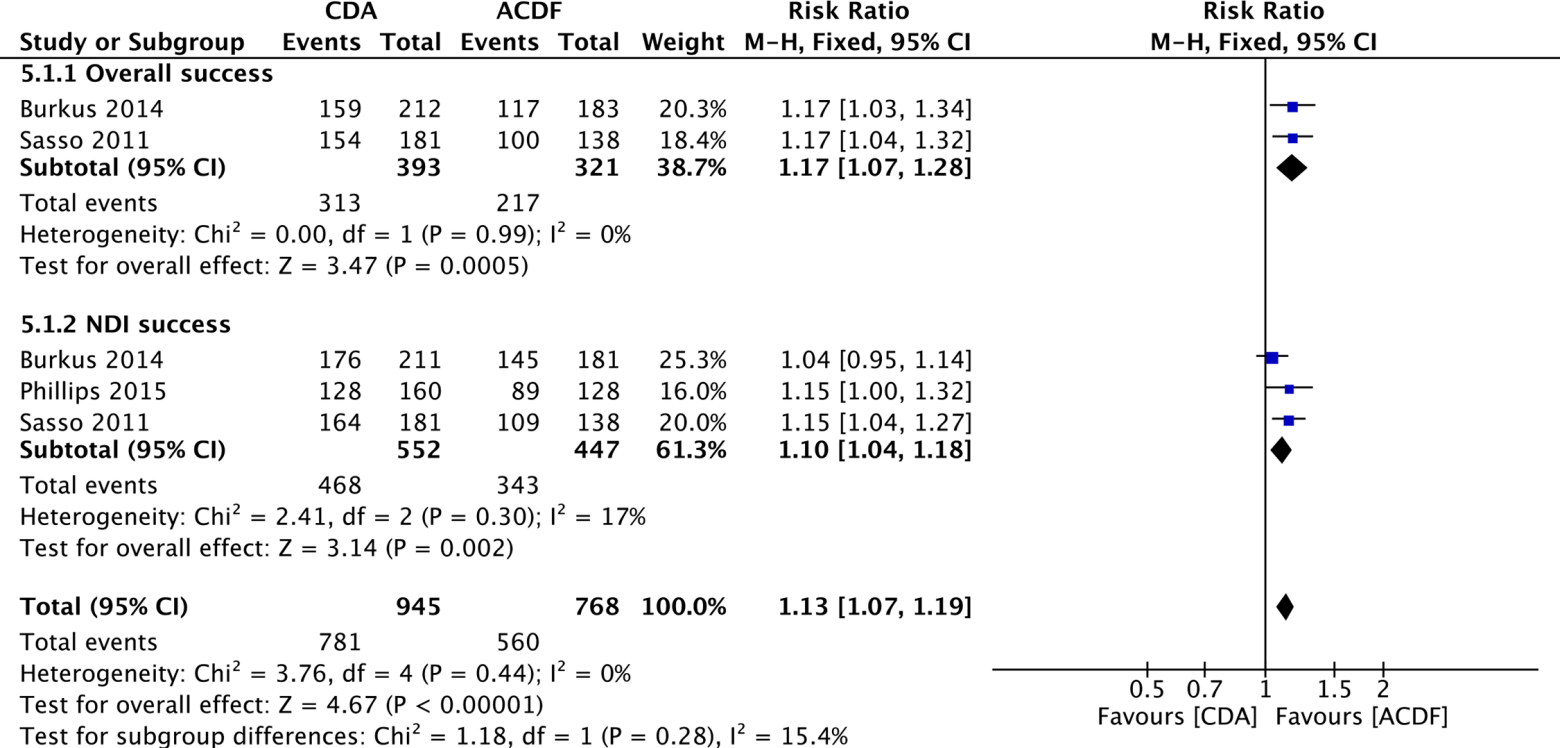
Forest plot for overall success and NDI success.

**Fig 3:**
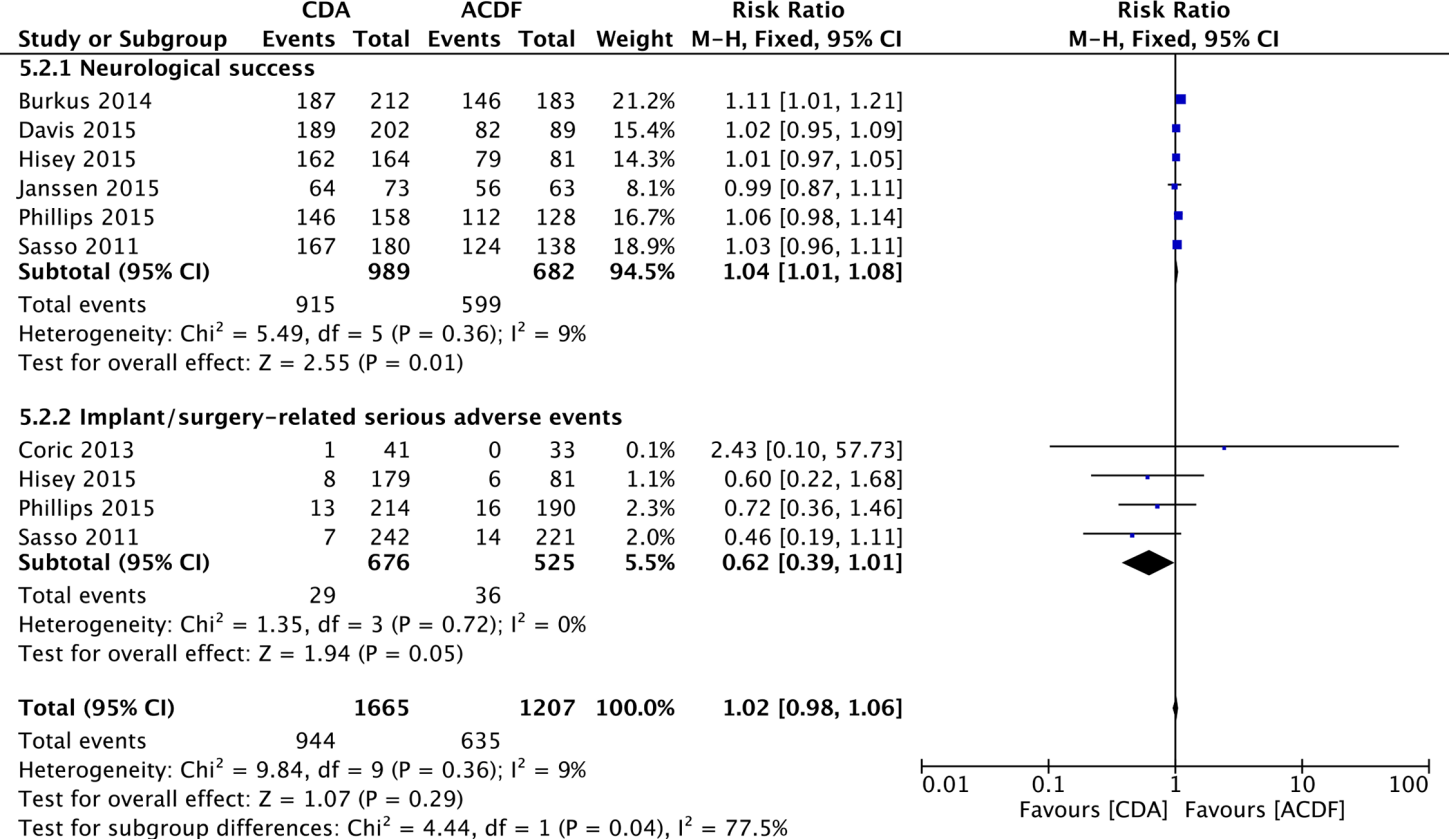
Forest plot for neurological success and implant/surgery-related serious adverse events.

Overall success was considered achieved if a patient met all of the following items: NDI success, neurological success, absences of implant/surgery-related serious adverse events and secondary procedure. Two studies [53, 58] reported the overall success data. Pooled analysis showed that patients in CDA group had a higher rate of overall success compared with that in ACDF group (RR = 1.17; 95%CI: 1.07, 1.28; P = 0.0005; I^2^ = 0%; Fig 2). NDI success was defined as postoperative NDI score improvement of at least a 15-point increase from preoperative score. Three studies [53, 57, 58] reported the NDI success data. Pooled analysis revealed a higher rate of NDI success in CDA group (RR = 1.10; 95%CI: 1.04, 1.18; P = 0.002; I^2^ = 17%; Fig 2). Neurological success was determined as postoperative maintenance or improvement in each of the individual neurological evaluations (muscle strength, sensory deficit, and reflex functions) compared with the preoperative status. Six studies [53,55–58,60] reported the neurological success data. Pooled analysis found a higher rate of neurological success in CDA group (RR = 1.04; 95%CI: 1.01, 1.08; P = 0.01; I^2^ = 9%; Fig 3).

### Implant/Surgery-Related Serious Adverse Events

Serious adverse events were defined as grade 3 or 4 adverse events based on the WHO criteria. Four studies [54,56–58] reported the data of implant/surgical procedure-related serious adverse events. Overall, there was a significant difference in favor of the CDA group (RR = 0.62; 95%CI: 0.39, 1.01; P = 0.05; I^2^ = 0%; Fig 3).

### Secondary Procedure

**Fig 4:**
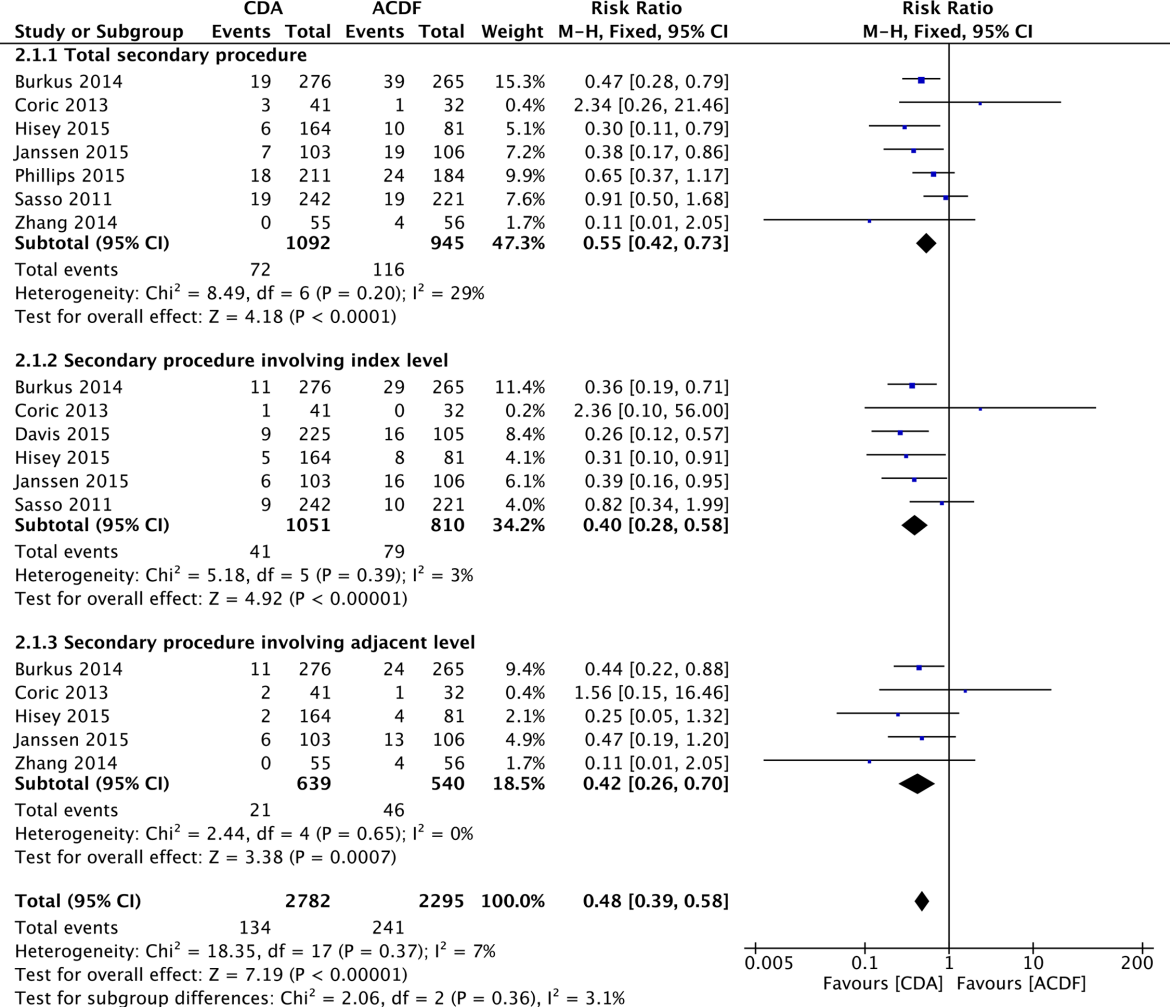
Forest plot for secondary procedure.

Secondary procedure was defined as any reoperation, revision, supplemental fixation, or implant removal. Total secondary procedure data were available in seven studies [53,54,56–60] and pooled analysis showed a lower rate in CDA group (RR = 0.55; 95%CI: 0.42, 0.73; P< 0.0001; I^2^ = 29%; Fig 4). Six studies [53–56,58,60] reported the data of secondary procedure involving the index level. Pooled analysis revealed a lower rate in CDA group (RR = 0.40; 95%CI: 0.28, 0.58; P< 0.00001; I^2^ = 3%; Fig 4). The data of secondary procedure involving the adjacent level were available in five studies [53,54,56,59,60]. Overall, the percentage of patients undergoing secondary procedure involving the adjacent level was lower in CDA group (RR = 0.42; 95%CI: 0.26, 0.70; P = 0.0007; I^2^ = 0%; Fig 4).

### Functional Outcomes

**Fig 5:**
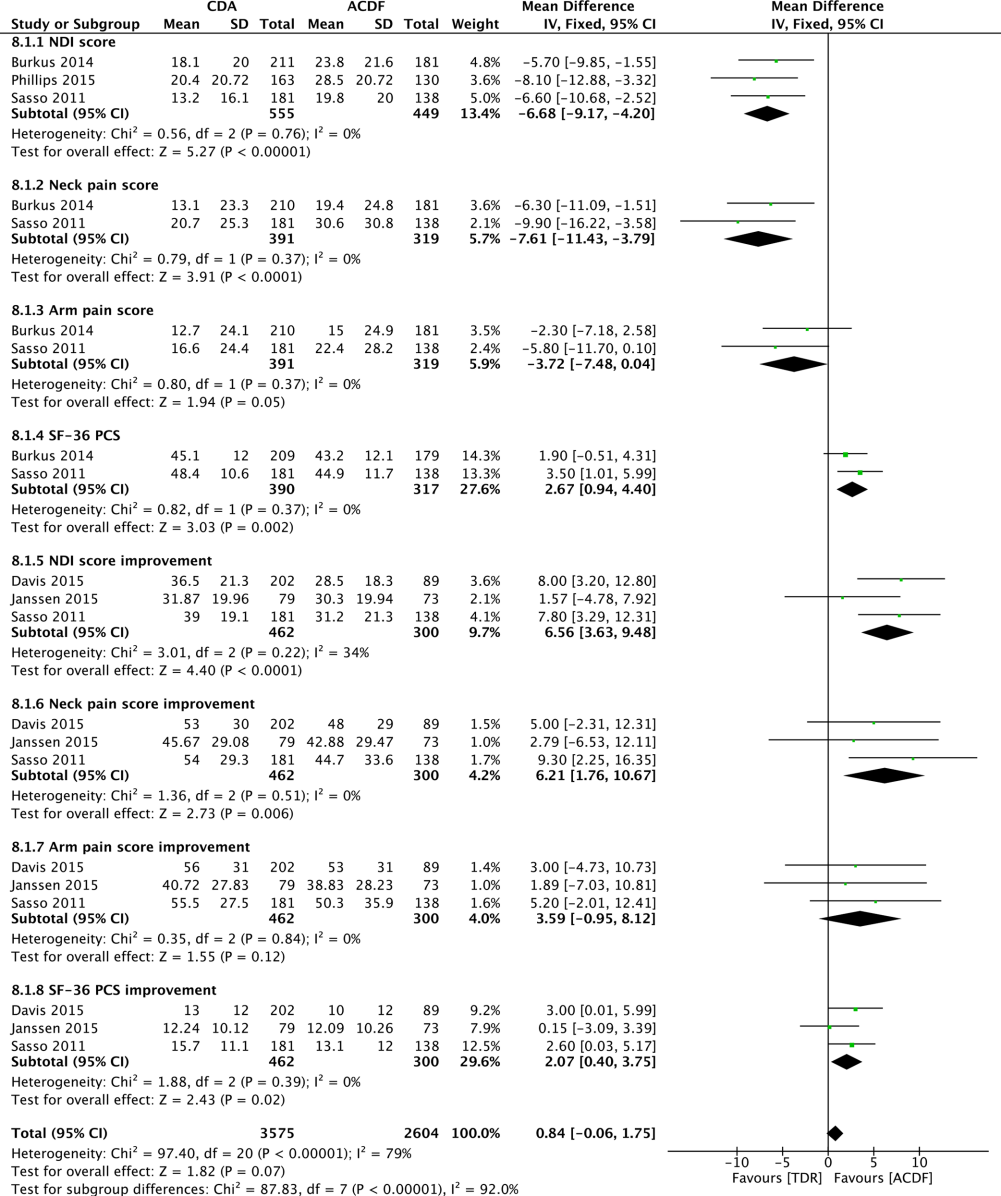
Forest plot for functional outcomes.

Three studies [53,57,58] reported the NDI score data. Pooled analysis indicated patients in CDA group had a better NDI score on last follow-up (WMD = -6.68; 95%CI: -9.17, -4.20; P< 0.00001; I^2^ = 0%; Fig 5). Neck and arm pain and SF-36 PCS were available in two studies [53,58]. Overall, patients in CDA group had a better neck pain score (WMD = -7.61; 95%CI: -11.43, -3.79; P< 0.0001; I^2^ = 0%; Fig 5), better arm pain score (WMD = -3.72; 95%CI: -7.48, 0.04; P = 0.05; I^2^ = 0%; Fig 5), and better SF-36 PCS (WMD = 2.67; 95%CI: 0.94, 4.40; P = 0.002; I^2^ = 0%; Fig 5). Three studies [55,58,60] reported the mean improvement from baseline through last follow-up in NDI score, neck and arm pain scores, and SF-36 PCS. Pooled estimate showed CDA group had greater improvement in NDI score (WMD = 6.56; 95%CI: 3.63, 9.48; P< 0.0001; I^2^ = 34%; Fig 5), neck pain score (WMD = 6.21; 95%CI: 1.76, 10.67; P = 0.006; I^2^ = 0%; Fig 5), and SF-36 PCS (WMD = 2.07; 95%CI: 0.40, 3.75; P = 0.02; I^2^ = 0%; Fig 5). In addition, CDA group had a greater improvement in arm pain score without statistical significance (WMD = 3.59; 95%CI: -0.95, 8.12; P = 0.12; I^2^ = 0%; Fig 5).

### Patient Satisfaction and Recommendation

**Fig 6:**
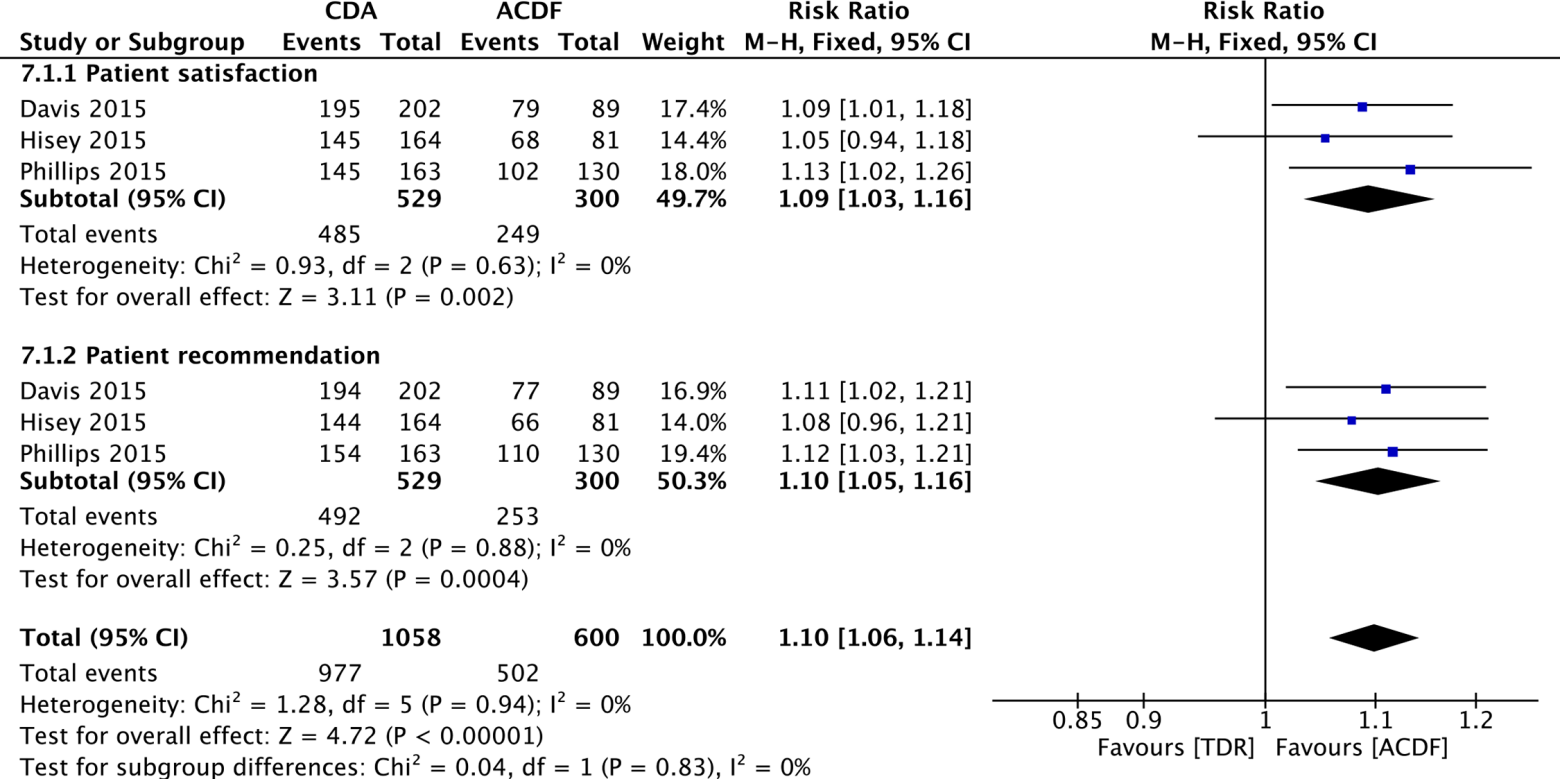
Forest plot for patient satisfaction and recommendation.

Three studies [55–57] reported the data about patient satisfaction and recommendation. The pooled results indicated that the percentage of patients satisfied with their treatment was higher in the CDA group (RR = 1.09, 95%CI: 1.03,1.16; P = 0.002; I^2^ = 0%; Fig 6). Pooled analysis showed a higher percentage of patients would recommend their treatment to a friend in CDA group (RR = 1.10; 95%CI: 1.05, 1.16; P = 0.0004; I^2^ = 0%; Fig 6).

### Radiological Adjacent Segment Degeneration

**Fig 7:**
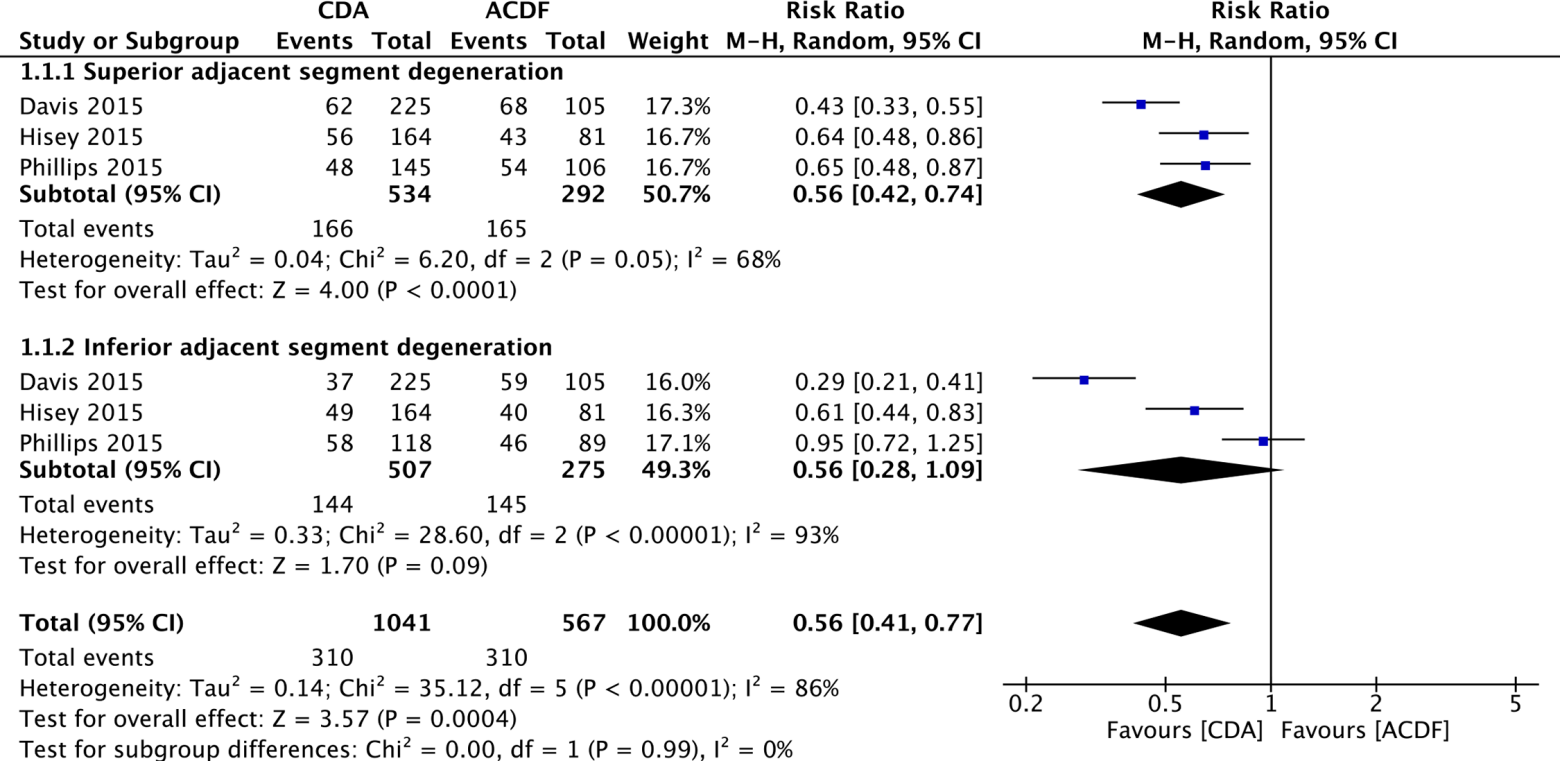
Forest plot for radiological adjacent segment degeneration.

Two studies [55,56] employed Kellgren-Lawrence scale [61] to assess adjacent segment degeneration. Pooled analysis of these two studies showed lower rates of adjacent segment degeneration in CDA group (total: 0.01; superior: P = 0.002; inferior: P = 0.02). One study [57] reported the incidence of adjacent segment degeneration determined by Walraevens’s grading system [62]. Their results showed there was significant difference between CDA and ACDF groups regarding superior adjacent segment degeneration (P = 0.004), but not inferior adjacent segment degeneration (P = 0.72) [57]. Pooled analysis of these three studies [55–57] revealed a lower rate of superior adjacent segment degeneration in CDA group (RR = 0.56; 95%CI: 0.42, 0.74; P< 0.0001; I^2^ = 68%; Fig 7). Regarding inferior adjacent segment degeneration, patients in CDA group had a lower rate without statistical significance (RR = 0.56; 95%CI: 0.28, 1.09; P = 0.09; I^2^ = 93%; Fig 7).

### Follow-Up Rate

**Fig 8:**
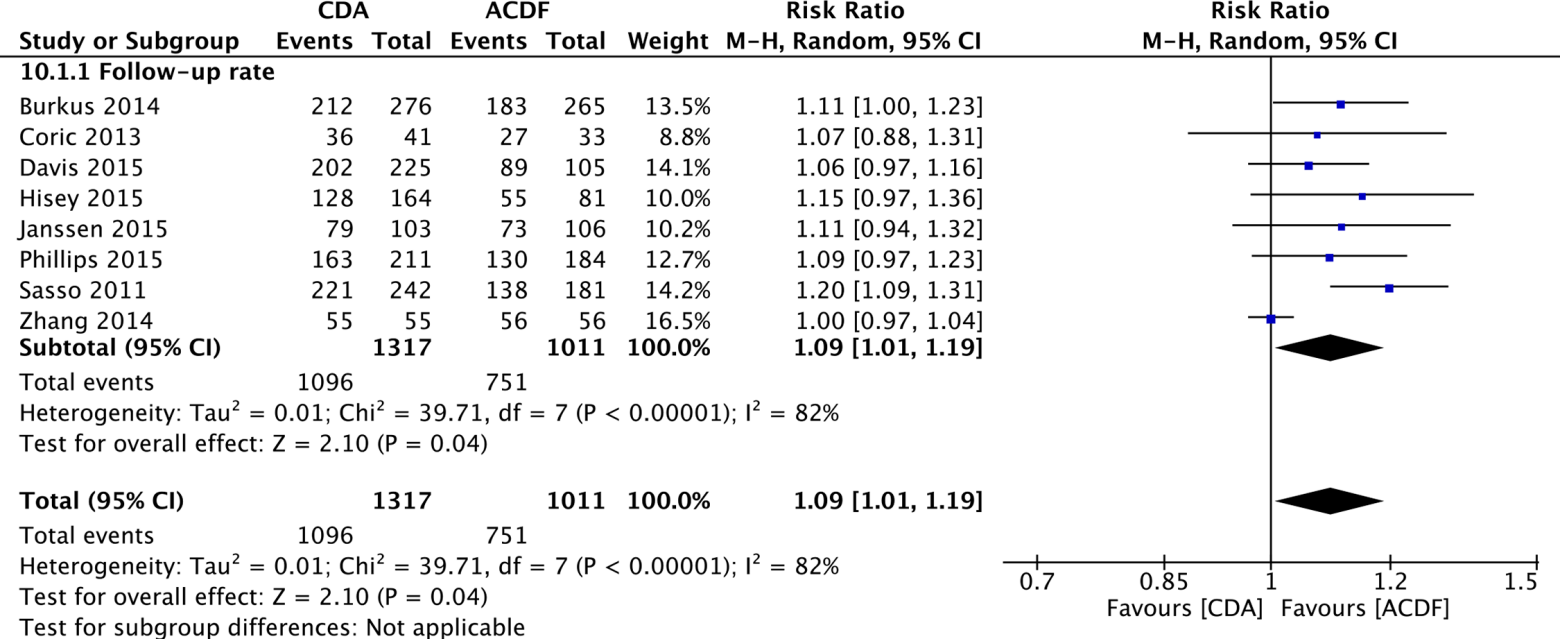
Forest plot for follow-up rate.

Pooled analysis of all studies found a significantly lower follow-up rate in ACDF group (RR = 1.09; 95%CI: 1.01, 1.19; P = 0.04; I^2^ = 82%; Fig 8). Only one study [59] did not have follow-up loss at last follow-up. Pooled analysis of seven studies with follow-up loss also found a significantly lower rate of follow-up rate in ACDF group (RR = 1.12; 95%CI: 1.07, 1.17; P< 0.00001; I^2^ = 0%).

### Subgroup Analysis

Subgroup analyses were performed on patients with only 1-level cervical disc disease. The results are shown in Table 3.

**Table 3:** Subgroup analysis of patients with 1-level cervical disc disease.

Outcomes	No. Studies	No. Patients	Statistical method	Effect estimate	P	X^2^	I^2^ (%)
**Overall success**	2	714	Risk Ratio (M-H, Fixed, 95% CI)	1.17 (1.07, 1.28)	0.0005	0	0%
**NDI success**	3	999	Risk Ratio (M-H, Fixed, 95% CI)	1.10 (1.04, 1.18)	0.002	2.41	17%
**Neurological success**	5	1380	Risk Ratio (M-H, Fixed, 95% CI)	1.05 (1.01, 1.09)	0.01	5.7	30%
**Implant/surgery-related serious adverse events**	4	1201	Risk Ratio (M-H, Fixed, 95% CI)	0.62 (0.39, 1.01)	0.05	1.35	0%
**Total secondary procedures**	7	2037	Risk Ratio (M-H, Fixed, 95% CI)	0.55 (0.42, 0.73)	< 0.0001	8.49	29%
**Secondary procedures involving index level**	5	1531	Risk Ratio (M-H, Fixed, 95% CI)	0.45 (0.30, 0.68)	0.0001	3.8	0%
**Secondary procedures involving adjacent levels**	5	1179	Risk Ratio (M-H, Fixed, 95% CI)	0.42 (0.26, 0.70)	0.0007	2.44	0%
**NDI score**	3	1004	Mean Difference (IV, Fixed, 95%CI)	—6.68 (-9.17, -4.20)	< 0.00001	0.56	0%
**Neck pain score**	2	710	Mean Difference (IV, Fixed, 95%CI)	—7.61 (-11.43, -3.79)	< 0.0001	0.79	0%
**Arm pain score**	2	710	Mean Difference (IV, Fixed, 95%CI)	—3.72 (-7.48, 0.04)	0.05	0.8	0%
**SF-36 PCS**	2	707	Mean Difference (IV, Fixed, 95%CI)	2.67 (0.94, 4.40)	0.002	0.82	0%
**NDI score improvement**	2	471	Mean Difference (IV, Random, 95%CI)	5.10 (-0.95, 11.15)	0.1	2.46	59%
**Neck pain score improvement**	2	471	Mean Difference (IV, Fixed, 95%CI)	6.93 (1.31, 12.55)	0.02	1.19	16%
**Arm pain score improvement**	2	471	Mean Difference (IV, Fixed, 95%CI)	3.89 (-1.71, 9.50)	0.17	0.32	0%
**SF-36 PCS improvement**	2	471	Mean Difference (IV, Fixed, 95%CI)	1.65 (-0.36, 3.67)	0.11	1.35	26%
**Patient satisfaction**	2	538	Risk Ratio (M-H, Fixed, 95% CI)	1.10 (1.02, 1.18)	0.02	0.91	0%
**Patient recommendation**	2	538	Risk Ratio (M-H, Fixed, 95% CI)	1.10 (1.03, 1.18)	0.006	0.24	0%
**Superior ASD**	2	496	Risk Ratio (M-H, Fixed, 95% CI)	0.65 (0.52, 0.80)	< 0.0001	0	0%
**Inferior ASD**	2	452	Risk Ratio (M-H, Random, 95% CI)	0.76 (0.49, 1.19)	0.24	4.44	77%

X^2^, chi-squared heterogeneity statistics; I^2^, index of heterogeneity; M-H, Mantel-Haenszel; CI, confidence interval.

## Discussion

Although artificial cervical discs have been utilized in spinal surgery for several years, ACDF remains the gold standard for the treatment of symptomatic cervical disc disease. It is partly attributable to the uncertainty of the long-term outcomes of cervical disc arthroplasty compared with the well-perceived long-term success of ACDF. To our knowledge, there are many meta-analysis studies available in the literature comparing the efficacy and safety of CDA with ACDF for the treatment of symptomatic cervical disc disease. However, most of them included the studies with short-term follow-up, which made it impossible to conclude the long-term comparativeness. Therefore, we performed a meta-analysis of eight RCTs with at least 48 months follow-up to determine whether CDA was superior over ACDF.

This meta-analysis found that patients in CDA group had a significantly higher overall success rate compared with that in ACDF group. Pooled analysis of NDI success and neurological success data also revealed to be in favor of CDA. Moreover, we extracted NDI, VAS, and SF-36 scores at last follow-up to evaluate functional outcomes. Pooled estimates of these data showed superiority in CDA except for arm pain score improvement data, which showed no significant difference. These findings suggested that CDA seemed to be more effective than ACDF for the treatment of cervical spondylosis.

Secondary procedure is an important clinical event with substantial clinical and financial burdens for the patient as well as additional cost for the payor. In this meta-analysis, we found that CDA was superior to ACDF regarding the rate of total secondary procedures. Pooled results of the data of secondary procedure involving index level or adjacent level also revealed superiority in CDA group. These results were consistent with Wu et al.’ findings [63]. However, they only included four randomized controlled trials with only 921 patients in total.

We observed that most of the adverse events reported in the included studies were medical problems unrelated to the index surgery or the cervical spine. Therefore, we chose implant/surgery-related serious adverse events for the assessment of safety. Pooled results showed a lower rate in CDA patients, suggesting CDA seemed to be surgically safer than ACDF for the treatment of symptomatic cervical disc disease. In addition, three studies reported the data of patient satisfaction and patient recommendation. Regarding these self-assessed data, this meta-analysis revealed better results reported in CDA patients, supporting the superior efficacy in CDA over ACDF.

Adjacent segment degeneration has been considered as one major concern for patients undergoing ACDF for degenerative disc disease [3–7]. Compared to cervical fusion, disc arthroplasty provides theoretical biomechanical advantage of motion preservation and stress reduction at adjacent levels [12–16]. However, it remains unclear whether CDA can decrease the incidence of adjacent segment degeneration compared to ACDF [33, 36, 40, 49, 64]. In this meta-analysis, no studies reported the rate of symptomatic adjacent segment disease while three studies reported the rate of radiological adjacent segment degeneration where pooled outcomes demonstrated a significantly lower rate of superior adjacent segment degeneration and an insignificantly lower rate of inferior adjacent segment degeneration in CDA patients. These findings suggested that CDA seemed to have positive effect on the process of adjacent segment degeneration. We noticed the statistical heterogeneity was high for these outcomes. This level of heterogeneity might be due to the difference of radiological criteria determined for adjacent segment degeneration and number of surgical levels. Of note, radiological adjacent segment degeneration is known to not directly correlate with symptomology [6,65]. Therefore, prospective RCTs with long-term follow-up reporting symptomatic adjacent segment disease as an outcome are warranted to clarify this question.

Several potential limitations should be acknowledged in our meta-analysis. First, only eight RCTs with follow-up between 4 to 7 years were included in this meta-analysis. Further studies with larger sample sizes and longer follow-up are warranted. Second, there were some methodological weaknesses in the included studies, such as unclear methods of allocation concealment and inadequate blinding procedures. Moreover, missing information such as the absence of ITT analysis and follow-up loss was presented in almost every study. All these methodological drawbacks would weaken the credibility of pooled outcomes. Third, patients undergoing an ACDF had a tendency to have poorer follow-up rate than those in CDA group (P = 0.04), which might lead to biased results. The reasons for this bias are not clear and probably multifactorial because of the lack of blinding of patients in all studies. Fourth, almost all the studies utilized a non-inferiority study design, which is typically less stringent in demonstrating efficacy than standard clinical trials. Despite these limitations, we still believe that this meta-analysis supports the superiority of CDA over ACDF on efficacy and safety for the treatment of symptomatic cervical disc disease in mid- to long-term follow-up.

## Supporting Information

S1 Fig Complete search strategy used in this study. (TIF)

S2 Fig PRISMA 2009 Checklist. (TIF)
