# Supplementary figures and images for: Mid- to Long-Term Outcomes of Cervical Disc Arthroplasty versus Anterior Cervical Discectomy and Fusion for Treatment of Symptomatic Cervical Disc Disease: A Systematic Review and Meta-Analysis of Eight Prospective Randomized Controlled Trials
Source: PLoS One. 2016 Feb 12;11(2):e0149312. doi: 10.1371/journal.pone.0149312 (PMC4752293; doi:10.1371/journal.pone.0149312)

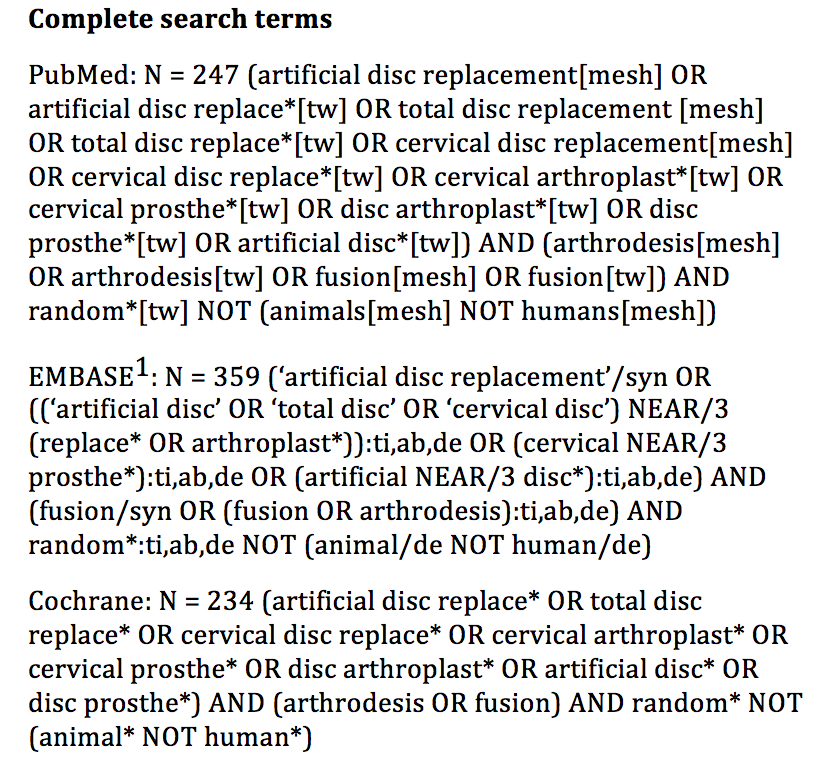

Supplement: S1 Fig — (TIF) [file pone.0149312.s001.tif]

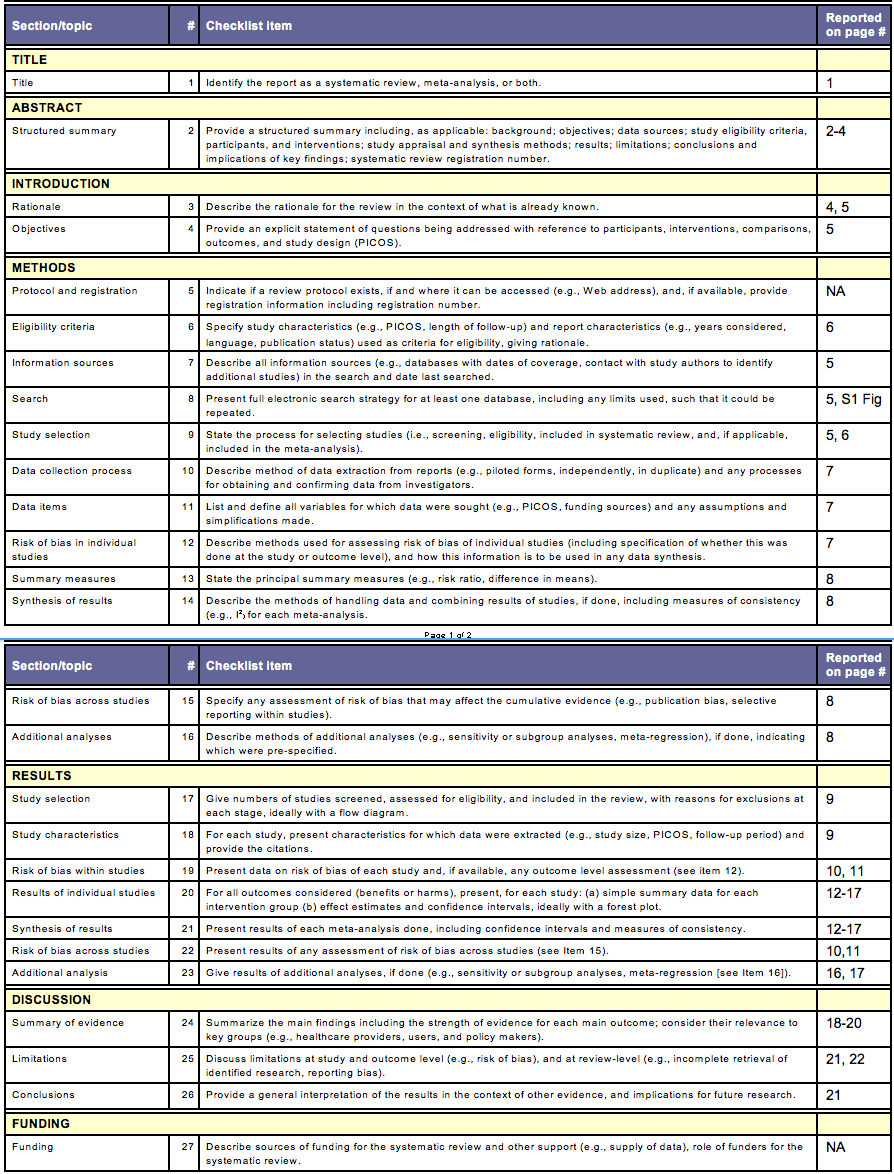

Supplement: S2 Fig — (TIF) [file pone.0149312.s002.tif]
